# Chemopreventive Property of Sencha Tea Extracts towards Sensitive and Multidrug-Resistant Leukemia and Multiple Myeloma Cells

**DOI:** 10.3390/biom10071000

**Published:** 2020-07-04

**Authors:** Xiaohua Lu, Mohamed E. M. Saeed, Mohamed-Elamir F. Hegazy, Christopher J. Kampf, Thomas Efferth

**Affiliations:** 1 Department of Pharmaceutical Biology, Institute of Pharmacy and Biochemistry, Johannes Gutenberg University, Staudinger Weg 5, 55128 Mainz, Germany; xiaohulu@uni-mainz.de (X.L.); saeedm@uni-mainz.de (M.E.M.S.); elamir77@live.com (M.-E.F.H.); 2 Chemistry of Medicinal Plants Department, National Research Centre, 33 El-Bohouth St., Dokki, Giza 12622, Egypt; 3 Department for Chemistry, Johannes Gutenberg University Mainz, Duesbergweg 10-14, 55128 Mainz, Germany; kampfc@uni-mainz.de

**Keywords:** green tea, catechins, chemotherapy, drug resistance, flavonoids, functional food, microarray analysis, natural products, polyphenols

## Abstract

The popular beverage green tea possesses chemopreventive activity against various types of tumors. However, the effects of its chemopreventive effect on hematological malignancies have not been defined. In the present study, we evaluated antitumor efficacies of a specific green tea, sencha tea, on sensitive and multidrug-resistant leukemia and a panel of nine multiple myelomas (MM) cell lines. We found that sencha extracts induced cytotoxicity in leukemic cells and MM cells to different extents, yet its effect on normal cells was limited. Furthermore, sencha extracts caused G2/M and G0/G1 phase arrest during cell cycle progression in CCRF/CEM and KMS-12-BM cells, respectively. Specifically, sencha-MeOH/H_2_O extracts induced apoptosis, ROS, and MMP collapse on both CCRF/CEM and KMS-12-BM cells. The analysis with microarray and COMPARE in 53 cell lines of the NCI panel revealed diverse functional groups, including cell morphology, cellular growth and proliferation, cell cycle, cell death, and survival, which were closely associated with anti-tumor effects of sencha tea. It is important to note that PI3K/Akt and NF-κB pathways were the top two dominant networks by ingenuity pathway analysis. We demonstrate here the multifactorial modes of action of sencha tea leading to chemopreventive effects of sencha tea against cancer.

## 1. Introduction

Tea, a product from leaf and bud of the plant *Camellia sinensis*, is the second most consumed beverage worldwide after water [1,2]. Today, over 30 countries are producing various kinds of tea varieties as a relaxation drink or for health benefits [3]. Tea can be classified into black tea, green tea, and oolong tea based on the fermentation degree [2,4]. Green tea with the most abundant polyphenols is non-fermented and produced by drying and steaming the freshly harvested leaves [1,5]. The health-promoting effects of green tea are mainly contributed to polyphenols, commonly known as catechins, which constitute up to 30% of fresh leaf dry weight [3]. The most prominent catechins are (-)-epigallocatechin-3-gallate (EGCG), (-)-epigallocatechin (EGC), (-)-epicatechin-3-gallate (ECG), (-)-epicatechin (EC), gallic acid (GA), (+)-catechin (C), (-)-gallocatechin (GC), and (-)-gallocatechin gallate (GCG) [6,7,8].

Increased consumption of tea catechins seems to provide more health benefits. A prospective cohort study reported that a lower risk of cancer incidence was observed for Japanese consuming over 10 cups a day versus those consuming below 3 cups [9]. The potential health benefits of tea catechins depend not only on the amount consumed but also on their bioavailability. Tea catechins are partly absorbed by intestines and excreted through bile, feces, and urine [10]. Although few publications have reported about the tissue distribution of tea catechins in humans, the absorption, distribution, and elimination of tea catechins have been reported on rats [11]. To date, green tea possesses not only anti-inflammatory and anti-oxidative but also anti-obesity, anti-diabetic, antimutagenic, and anticarcinogenic biological properties [1,2]. Recent human studies suggest that green tea may reduce the risk of medical chronic conditions such as cancer, cardiovascular disease, and diabetes [3,12]. Consumption of green tea has been linked to preventing various types of solid cancer [13,14,15,16].

Green tea inhibits not only the initiation and promotion of the carcinogenic process but also progression [17,18,19,20,21]. Tea catechins are generally considered to be responsible for anticancer and therapeutic properties due to their radical scavenging and metal chelating functions, as well as anti-mutagenic activities [19,22]. Despite tea catechins being able to exert its actions by serving as an antioxidant or pro-oxidant, the overwhelming majority of studies reported that tea catechins can either be oxidized to generate reactive oxygen species (ROS) or produce ROS after entering cells to kill cancer cells [17,23,24]. Mitochondria are the major sites where ROS are produced and the apoptotic machinery is activated [25]. High levels of ROS within mitochondria can cause oxidative DNA damage, activate oncogenes, and initiate apoptosis by opening mitochondrial membrane potential (MMP) [26]. Thus, one of the early processes that leads to the final stage of apoptosis can be observed from the collapse of mitochondrial membrane potential MMP [25]. Additionally, a significant result revealed that green tea catechins differentially caused cell cycle arrest and induction of apoptosis by the upregulation of p21/waf1 and Bax proteins and downregulation of histone deacetylases [27]. The major catechin of green tea promotes apoptosis in cancer cells via modulation of the phosphoinositide 3-kinase (PI3K)/Akt pathway, Bcl-2 family proteins, HIF-1α as well as nuclear factor κB (NF-κB) [28,29,30]. These investigations clearly demonstrate that green tea prevents cancer through multiple molecular mechanisms.

Acute lymphoblastic leukemia (ALL) and multiple myeloma (MM) are two common hematologic malignancies. With an estimated 6590 new cases and over 1400 deaths in 2016 alone, ALL is the second most common acute leukemia in the US [31]. MM still constitutes about 15% of annual reported cases of hematological malignancies in the Western world [32,33,34]. Despite the continuous development of medical treatment, ALL and MM have still presented poor survival outcomes owing to limitations of current cancer therapies (e.g., drug resistance and severe side effects) [35,36,37]. The overexpression of the ATP-binding cassette (ABC) transporter proteins, especially P-glycoprotein (P-gp, ABCB1/MDR1), confer most prominent multidrug resistance [38]. Overexpression of P-gp has been reported on ALL patients [39]. Severe heart failure and pulmonary complications may be observed on MM patients after the targeted therapy of bortezomib [40,41]. It is documented that tea catechins, both EGCG and EGC, showed a sensitizing effect of doxorubicin on drug-resistant cell lines [42].

Although green tea has been well investigated in various types of solid cancers, it is still largely unknown, whether or not green tea reveals chemopreventive activity against P-gp resistant acute leukemia cells and MM cells. To date, the role of green tea in hematologic malignances is poorly understood. We previously demonstrated that both phenolic and non-phenolic fractions of green tea inhibited the migration of human HepG2 hepatocellular carcinoma [18]. However, our current study has been designed to investigate the effects of green tea extracts on sensitive and P-gp overexpression resistant leukemia cells as well as a range of nine MM cells in vitro. Most importantly, for the systematic and comprising search to reveal the multifactorial modes of action of green tea, we have also performed microarray and COMPARE analyses of EGC in 60 cell lines of the National Cancer Institute (NCI) panel.

## 2. Materials and Methods

### 2.1. Chemicals and Extracts

Certified USDA organic sencha powder was purchased from Sei Mee Tea^®^ LLC. Sencha powder (around 3.0 g) were extracted with different solvents as the following: methanol (MeOH), MeOH: H_2_O (7:3), and H_2_O. Then, the extracts were concentrated under vacuum to afford darkish brown gum. The extraction yields of sencha-MeOH (70% MeOH/H_2_O) extracts were 32.01%, 35.01%, and 31.71%, respectively. The dry extracts were kept at −20 °C for HPLC-HRMS/MS as well as biological activity analyses. Sencha extracts were prepared as a stock solution (20 mg/mL) in DMSO. Reference compounds—including EGCG (purity ≥ 99%, CAS989-51-5), GCG (purity ≥ 99%, CAS4233-96-9), GA (purity ≥ 99%, CAS5995-86-8), ECG (purity ≥ 99%, CAS1257-08-5)—were purchased from Tokyo Chemical Industry Co., Ltd (Tokyo, Japan).

### 2.2. Extract Analyses by HPLC-HRMS/MS

Dried extracts were re-dissolved in MeOH and/or MeOH: H_2_O (7:3) at a concentration (1:1000) of 2 mg/mL and diluted by a factor of 1000, followed by analysis using a 1260 Infinity II high-performance liquid chromatography (HPLC) system (Agilent Technologies, Waldbronn, Germany) coupled to a 6545 QTOF mass spectrometer (Agilent Technologies, Waldbronn, Germany) equipped with an Agilent Jet Stream electrospray ionization (ESI) interface. Chromatographic separation was performed on an EclipsePlus C18 RRHP (50 mm × 2.1 mm, 1.8 μm, Agilent Technologies, Waldbronn, Germany) column eluted with the following gradient of 2% acetonitrile in H_2_O (A) and methanol (B): 15% B for 1 min, linear gradient to 95% B in 24 min, 95% B for 5 min, back to 15% in 1 min, and re-equilibration at 15% B for 9 min. The flow rate was 0.2 mL/min. Analyses of the extract samples (5 μL injection volume) were performed in positive ion automated data-dependent acquisition mode, in which full mass spectrometry (MS) scans from m/z 50–1500 Da are acquired as MS1 survey scan (scan rate: 1 spectrum/s) and then MS/MS scans for the two most intense ions follow. MS/MS acquisition was set to be the active exclusion of precursors after 5 spectra for 0.5 min. The ESI conditions were set as follows: capillary voltage 3.5 kV, nozzle voltage 1 kV, fragment 175 (arbitrary units), drying gas temperature 320 °C, sheath gas temperature 350 °C, drying gas flow 10 L/min, and nebulizer pressure 35 psig. High-purity nitrogen was used as the nebulizer, auxiliary gas, and collision gas. Data were acquired in centroid mode, and the instrument was calibrated directly before each set of samples. The collision energy was fixed at 35 V.

### 2.3. Molecular Network Analyses

The HPLC-HRMS/MS were converted to universal mzXML format files using MSConvert, part of the ProteoWizard package, followed by uploading converted files to GNPS for molecular networking. The molecular networks were created using the online workflow of the Global Natural Products Social (GNPS) molecular networking platform (http://gnps.ucsd.edu). MS/MS spectra were filtered by a clustering algorithm with a cosine score of 0.7 and a minimum of 6 matched peaks. The resulting spectral network was imported into Cytoscape version 3.7.1. The nodes related to solvent (blank) were subtracted and the remaining nodes represented parent (+) m/z of metabolites detected in analyzed extracts as well as the authentic samples to indicate the similarity with tested extracts.

### 2.4. Cell Culture

Two leukemia cell lines, CCRF/CEM (drug-sensitive) and CEM/ADR5000 (multidrug-resistant), and 9 MM cell lines were used, including MOLP-8, RPMI-8226, NCI-H929, KMS-12-BM, KMS-11, OPM-2, AMO-1, L-363, and JJN-3. These cells were routinely cultured in RPMI 1640 medium (Sigma-Aldrich, Taufkirchen, Germany), supplemented with 10% fetal bovine serum (Invitrogen, Darmstadt, Germany) and 1% penicillin/streptomycin (Invitrogen). The details about the cell lines were described previously by our group [43]. Moreover, doxorubicin (5000 ng/mL) was used to maintain the resistance of CEM/ADR5000 cells [44]. Doxorubicin was kindly provided by the University Medical Center, Johannes Gutenberg University (Mainz, Germany). Bortezomib, the positive drug, was purchased from LC Laboratories (Massachusetts, MA, USA).

### 2.5. Resazurin Assay

The resazurin-based cytotoxicity assay was previously described [22,23]. This assay is based on the metabolic reduction of the non-fluorescent dye resazurin to highly fluorescent resorufin by viable cells [45]. The resazurin reduction assay was performed to assess the cytotoxicity of sencha-MeOH, -MeOH: H_2_O (7:3), -H_2_O extracts towards the selected cells [46]. All IC_50_ values were expressed as the mean ± standard deviation (SD). The experiments were repeated three times independently with six replicates for each concentration.

### 2.6. Cell Cycle Analysis and Detection of Apoptotic Cells by Flow Cytometry and Annexin V/PI Staining

One million of CRF/CEM cells were indeed in a 6-well plate and treated with sencha-MeOH/H_2_O extract, doxorubicin (the reference drug) or DMSO (used as solvent control) at four different concentration of 0.5 × IC_50_, 1 × IC_50_, 2 × IC_50_, and 4 × IC_50_. The same number of KMS-12-BM cells were also treated with sencha extracts, bortezomib (the reference drug), or DMSO. The corresponding IC_50_ values were based on the results of the resazurin assay (see above). After 24 h, 48 h, and 72 h incubation (humidified 5% CO_2_, 37 °C), cells were washed with cold phosphate-buffered saline (PBS) and then fixed with cold 80% ethanol. Cells were kept at −20 °C for 24 h. After washing with cold PBS, cells were re-suspended in cold PBS with 20 ug/mL RNAse for 1 h at room temperature and then stained with 50 μg/mL propidium iodide (PI) (Thermo Fisher Scientific, Darmstadt, Germany). Cell cycle analysis was performed using the BD Accuri^TM^ C6 Flow Cytometer (BD Biosciences, Heidelberg, Germany) [47,48,49,50]. Cell cycle data were analyzed with ModFit LT™.

One million of CCRF/CEM cells were treated with sencha-MeOH and H_2_O extract, doxorubicin, or DMSO at various concentrations. The same amounts of KMS-12-BM cells were also treated with the two sencha extracts, bortezomib or DMSO. After 48 h incubation under the normal condition, apoptosis was further assessed using fluorescein isothiocyanate (FITC)-conjugated annexin V/propidium iodide (PI) assay kit (eBioscience^TM^ Annexin V; Invitrogen, San Diego, CA, USA) using the BD Accuri^TM^ C6 Flow Cytometer. Annexin V/PI staining is a typical method to detect cells being early (FITC^+^/PI^-^) and late apoptosis (FITC^+^/PI^+^) as well as in a necrotic stage (FITC^+^/PI^+^) [51,52]. Early and late apoptosis/necrosis was evaluated on fluorescence 3 (FL3 for PI) versus fluorescence 1 (FL1 for annexin V) plots.

### 2.7. Measurements of ROS

One million of CCRF/CEM or KMS-12-BM cells were exposed to sencha-MeOH and -H_2_O extracts, a solvent control (DMSO), or a positive control hydrogen peroxide (H_2_O_2_) in a 6-well plate for 48 h. Then, the measurement of ROS production was evaluated with the cell-permeable and non-fluorescent 2′,7′-dichlorodihydrofuorescein diacetate (H2DCFHDA) (Sigma-Aldrich, Taufkirchen, Germany), which could be converted to the highly fluorescent 2′,7′-dichlorofluorescein (DCF) by intracellular hydroxyl and peroxyl. Cells were measured with BD Accuri^TM^ C6 Flow Cytometer using FL-1 the channel (488 nm excitation) [48,53]. 10^4^ events were counted for each sample. Mean FL-1 A of control and treated cells was used for analysis. 

### 2.8. Assessment of MMP

Aliquots of 10^5^ cells (CCRF/CEM or KMS-12-BM)/well were seeded in 96-well flat-bottom plate in a volume of 200 µL and treated with DMSO as a negative control, doxorubicin/ bortezomib or 0.5-, 1-, or 2-, 4-fold IC_50_ of sencha-MeOH and -H_2_O extracts for 24 h. JC-1 Mitochondrial Membrane Potential Assay Kit (Cayman Chemical, Ann Arbor, Michigan, USA) was used to detect MMP by flow cytometry according to the manufacturer’s instructions. Changes in MMP were analyzed using a BD LSRFortessa SORP equipped with five lasers lines. A yellow-green (561 nm) laser was used to excite and detect J-aggregates (live cells) and emitted light was collected using a 586/15 bandpass filter. Monomeric JC-1 (dead cells) was excited with a blue laser (488 nm) and emitted light was collected using a 530/30 bandpass filter [54]. Cells were first gated according to forward (F-) and side (S-) scatter properties. Doublets were removed using the area (A) and width (W) properties of the FSC (FSC-A/FSC-W). 10^4^ events from the FSC/SSC gate were recorded from each well. Data were analyzed using FlowJo V10.6.2 (BD Biosciences, Heidelberg, Germany).

### 2.9. COMPARE and Hierarchical Cluster Analyses of Microarray Data

A panel of 53 human tumor cell lines from the NCI (http://dtp.nci.nih.gov) was used to perform COMPARE analyses. The mRNA microarray hybridization of the NCI human tumor cell lines and logarithmic IC_50_ values (log_10_IC_50_) of these cell lines towards EGC have been deposited at the NCI website [55]. COMPARE analysis was an approach to producing the rank-ordered lists of genes expressed in the NCI cell lines [56]. Pearson’s rank correlation coefficients (R-values) were generated from log_10_IC_50_ values of EGC and microarray-based mRNA expression values to derive the COMPARE ranking. Nowadays, COMPARE analysis has developed as a standard procedure to identify candidate genes for drug resistance and sensitivity [57,58].

Hierarchical cluster analyses are a method to group heterogeneous objects into clusters of homogeneous objects. All objects are assembled into a cluster tree (dendrogram). The shorter the branch distance, the closer degree relatedness. Thus, objects with closely related features exhibited tightly together, while the separation in the cluster tree increases with progressive dissimilarity [59,60]. We clustered the mRNA expression of genes identified by COMPARE analyses using the CIMMINER program (https://discover.nci.nih.gov/cimminer/). One matrix clustered image (CIM) map and the Ward method were performed. The heat-map was accordingly obtained. To determine the significant difference between the distribution of sensitive or resistant cell lines, the chi-square (χ^2^) test was performed using the SPSS software program (version 21.0; SPSS Inc., Chicago, IL, USA). 

### 2.10. Ingenuity Pathway Analysis (IPA)

Ingenuity pathway analysis (IPA, http://www.ingenuity.com/) has been developed to predict downstream effects and interpret the relationship between the set of molecules involves and identify the most significant pathways [61]. IPA has been applied to find out genetic aberrations and the key pathways in major depressive disorder and breast cancer [62,63]. In the present study, those genes that were identified by COMPARE analysis to determine cellular responsiveness towards EGC were subjected to IPA (Qiagen Bioinformatics, Redwood City, CA, USA). Core analyses were further carried out to interpret these genes in the context of canonical pathways, diseases, functions, and relevant networks [64].

### 2.11. Statistics

Data were obtained from three independent experiments and expressed as mean ± standard deviation (SD). Student’s *t*-test or one-way analysis of variance (ANOVA) followed by LSD test was used for statistical analysis by SPSS software program (version 21.0; SPSS Inc., Chicago, IL, USA). The differences were considered statistically significant when *p* < 0.05.

## 3. Results and Discussion

### 3.1. Molecular Network Analyses for HPLC–HRMS/MS Data

To date, the quantitative and qualitative analysis of the major catechins in green tea has been well studied by UPLC-UV or HPLC/MS method [6,8,18]. In this study, the sencha-MeOH, -70% MeOH, -H2O extracts and authentic samples (including EGC, EGCG, GCG, ECG, and GA) were analyzed by HPLC-HRMS/MS. The contents of EGC, EGCG, GCG, ECG, and GA were expected as 0.06%, 18.36%, 0.16%, 0.12%, and 0.29% in sencha-MeOH extract; 26.3%, 13.28%, 0.0058%, 0.47%, and 0.022% in sencha-70% MeOH extract; 0.40%, 4.90%, 0.034%, 0.0066%, and 0.048% in sencha-H_2_O extract, respectively, indicating EGC and EGCG are the major compounds in sencha extracts.

**Figure 1:**
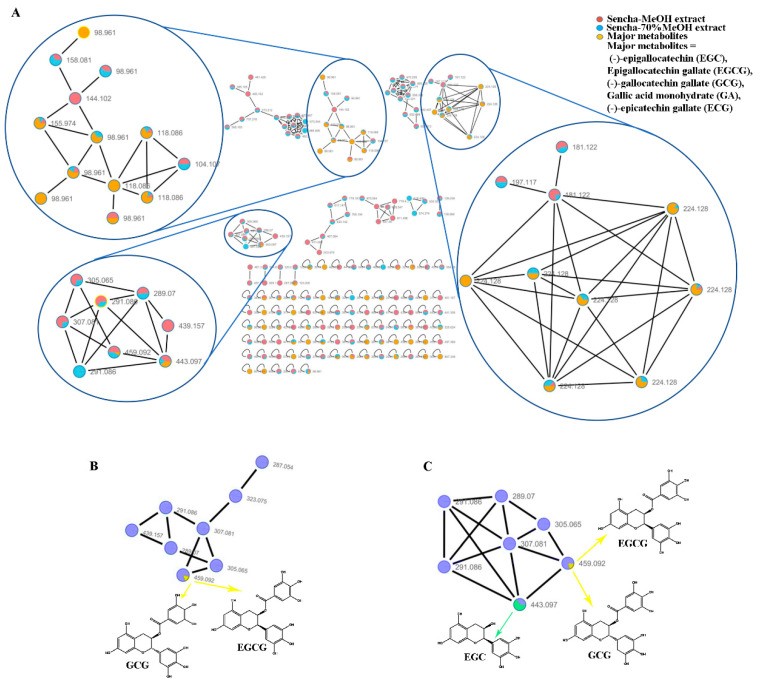
Molecular network analyses for HPLC–HRMS/MS data of sencha extracts by GNPS and Cytoscape. Spectral features of the major catechins in sencha extracts (**A**), GNPS molecular network of the major catechins in sencha-MeOH extract (**B**), and sencha-70% MeOH (**C**).

A massive amount of detailed information on the chemical composition of crude extracts can be generated from HPLC-HRMS/MS. The integration of molecular networking (MN) and in-silico fragmentation tools have been recently proposed as a powerful tool to provide a new perspective for early metabolite identification in natural product research [65]. To further find out about the relative abundance of a molecule in extracts, all converted data with mzXML format were analyzed using GNPS online tools to cluster similar spectra based on molecular weight. The results were visualized using Cytoscape V 7.3.1 with node pies in the force-directed layout. The spectral features indicated that both sencha-MeOH and -70% MeOH extracts shared the major authentic compounds: EGC, EGCG, GCG, ECG, and GA (Figure 1A). However, these compounds in sencha-H_2_O extract were hardly to plot (data not show), which may result from the low concentration. The sencha-MeOH and sencha-70% MeOH extracts were reanalyzed on the authentic compounds by the GNPS molecular network. By comparison of MS/MS reference samples with prepared extracts, the molecular networks showed the presence of EGCG/GCG as major compounds for both sencha-MeOH and sencha-70% MeOH extracts (Figure 1B,C). Additionally, the MS/MS comparison for sencha-70% MeOH extract indicated that EGC is the most abundant compound.

### 3.2. Resazurin-Based Cytotoxicity of Sencha Extracts

**Figure 2:**
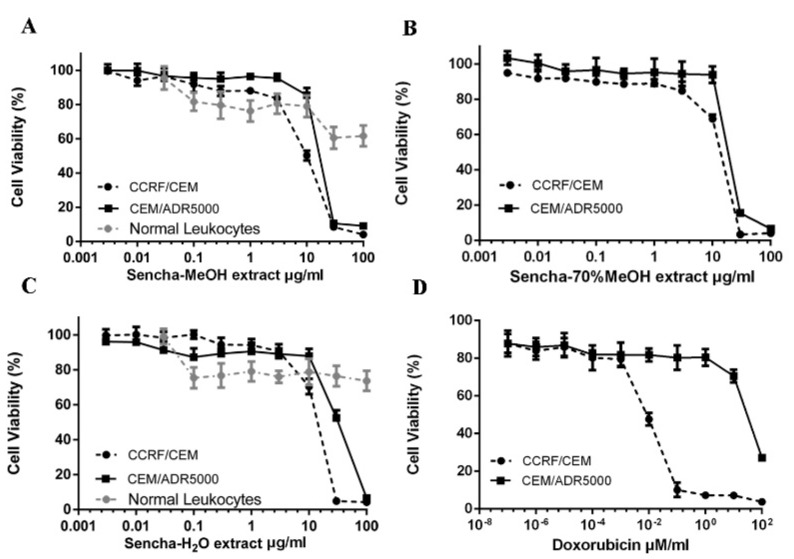
Growth inhibition of CCRF/CEM and P-gp-expressing CEM/ADR5000 leukemia cell lines towards three different extracts of sencha tea and doxorubicin at different concentrations. (**A**) Sencha-MeOH extract, (**B**) Sencha-70% MeOH extract, (**C**) Sencha-H_2_O extract, (**D**) doxorubicin.

To study the cytotoxic effects of the three sencha extracts, sensitive CCRF/CEM and P-gp-expressing CEM/ADR5000 leukemia cell lines were treated with three sencha extracts for 72 h up to the highest concentration of 100 μg/mL. All the three sencha extracts did not show significant cytotoxicity up to 8 μg/mL. The sencha-MeOH extract introduced cytotoxicity towards both cell lines with IC_50_ values of 8.38 ± 0.72 μg/mL and 18.52 ± 1.98 μg/mL, respectively (Figure 2A and Table 1). The IC_50_ values of sencha-70% MeOH extract on sensitive and resistant cells were 11.34 ± 1.86 μg/mL and 21.57 ± 2.69 μg/mL, respectively (Figure 2B). Additionally, CCRF/CEM cells were sensitive towards the sencha-H_2_O extract with an IC_50_ value of 11.5 ± 1.3 μg/mL, smaller than that of CEM/ADR5000 cells (33.8 ± 3.55 μg/mL) (Figure 2C). Considering the percentage of EGC (0.06%) and EGCG (18.36%), the equivalent content of EGC and EGCG were 0.005 and 1.54 μg/mL for sencha-MeOH extract towards CCRF/CEM cells. Doxorubicin, a substrate of P-gp, was used as a control drug. It revealed IC_50_ values of 0.0093 ± 0.00 μM in sensitive and 72.43 ± 2.61 μM in resistant cells (Figure 2D). As indicated by the degrees of cross-resistance, doxorubicin was remarkably more active on sensitive cells than on resistant ones, while sencha extracts showed more sensitivity to sensitive cells than resistant cells with the degree of resistance at 2.21, 1.90, and 2.94 for sencha-MeOH, -70% MeOH and -H_2_O extracts, respectively. It is worth mentioning that sencha tea affected normal lymphocytes less than sensitive and resistant cells.

**Figure 3:**
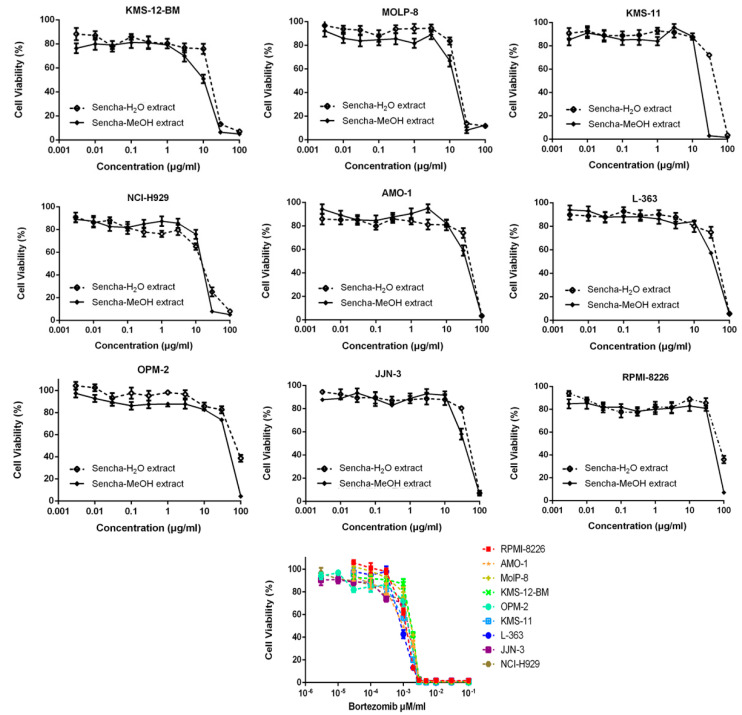
Growth inhibition of 9 MM cell lines towards sencha-MeOH, -H_2_O extracts, and bortezomib at different concentrations.

Sencha-MeOH extract showed the strongest inhibition for sensitive CCRF/CEM cells among the three extracts. Sencha tea represents an aqueous decoction and experimental validation of H_2_O extracts may be closer to the daily use of sencha tea. Thus, the cytotoxicity of sencha-MeOH and -H_2_O extracts was further studied on 9 MM cell lines. The sencha-MeOH extract inhibited the growth of MM cells with a stronger inhibition than sencha-H_2_O extract. The IC_50_ values varied from 11.37 (± 1.03) to 68.90 (± 6.25) µg/mL and 14.85 (± 1.44) to 76.63 (± 1.36) µg/mL for sencha-MeOH and -H_2_O extracts, respectively (Figure 3 and Table 2). KMS-12-BM cells showed the highest sensitivity among the 9 MM cell lines towards sencha extract. Considering the percentage of EGC (0.06%) and EGCG (18.36%), the equivalent content of EGC and EGCG were 0.0068 and 2.09 μg/mL for sencha-MeOH extract towards KMS-12-BM cells. The mechanisms by which sencha tea exerts cytotoxicity on acute leukemia and MM cells warrant further investigation.

### 3.3. Cell Cycle Analysis

**Figure 4:**
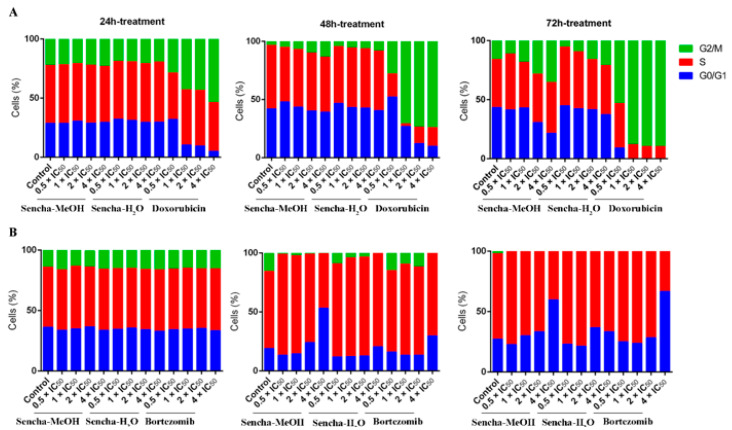
Distribution of cell cycle of CCRF/CEM (**A**) and KMS-12-BM (**B**) upon 24 h, 48 h, and 72 h treatment with sencha-MeOH extract, sencha-H_2_O extracts, doxorubicin, or bortezomib at a set of concentrations. IC_50_ values were 8.38 μg/mL for sencha-MeOH extract, 11.50 μg/mL for sencha-H_2_O extract, 0.0093 μM for doxorubicin towards CCRF/CEM cells. IC_50_ values were 11.37 μg/mL for sencha-MeOH extract, 14.85 μg/mL for sencha-H_2_O extract, 0.0019 μM for bortezomib towards KMS-12-BM cells.

Cancer is characterized by uncontrolled tumor cell proliferation and aberrant cell cycle progression [66]. Since we observed the growth inhibition of sensitive leukemia cells and a panel of MM cell lines, we would like to further investigate which phase of the cell cycle was arrested. The distribution of the cell cycle was analyzed on the most sensitive cells CCRF/CEM and KMS-12-BM by flow cytometer. CCRF/CEM cells showed a clear arrest at the G2/M phase after 48 h and 72 h treatment, although no obvious cell cycle arrest was observed after 24 h incubation (Figure 4A and Supplementary Materials Figures S1–S3). Interestingly, cell cycle arrest at the G2/M phase was observed only at a higher concentration of sencha-H_2_O extract (4 × IC_50_). Tea catechin EGCG was also reported to inhibit the proliferation of intestinal epithelial cells (colon carcinogenesis) but block cell cycle transition at the G1 phase, which likely occurs through suppression of cyclin D1 expression [67]. Doxorubicin, as the positive control, could induce G2/M-arrest (Figure 4A), consistent with our previous report [54].

Green tea seems to induce cell cycle arrest at different phases for different cancer cells. Cell cycle arrest in the G0/G1 phase was reported on prostate cancer cells and carcinoma cells upon green tea or its major catechins treatment [27,68]. This was also observed on KMS-12-BM cells, which showed a clear arrest in the G0/G1 phase of the cell cycle upon treatment for 48 h with 4-fold IC_50_ of sencha-MeOH extract (Figure 4B and Supplementary Materials Figures S4–S6), accompanied by a slight increase in the proportion of S phase percentage at a lower concentration of sencha-MeOH extract (0.5, 1 × IC_50_). After 72 h treatment, sencha-MeOH extract induced a higher extent of G0/G1 phase arrest than sencha-H_2_O extract. The positive drug bortezomib could also arrest cells at G0/G1 (Figure 4B). Both sencha-MeOH and sencha- H_2_O extracts caused sub-G0/G1 induction after 48 h exposure (Supplementary Materials Figures S2 and S5), indicating the induction of apoptosis. Thus, these results showed that sencha tea induced G2/M or G0/G1 phase arrest followed by apoptosis.

### 3.4. Induction of Apoptosis by Sencha Extracts

**Figure 5:**
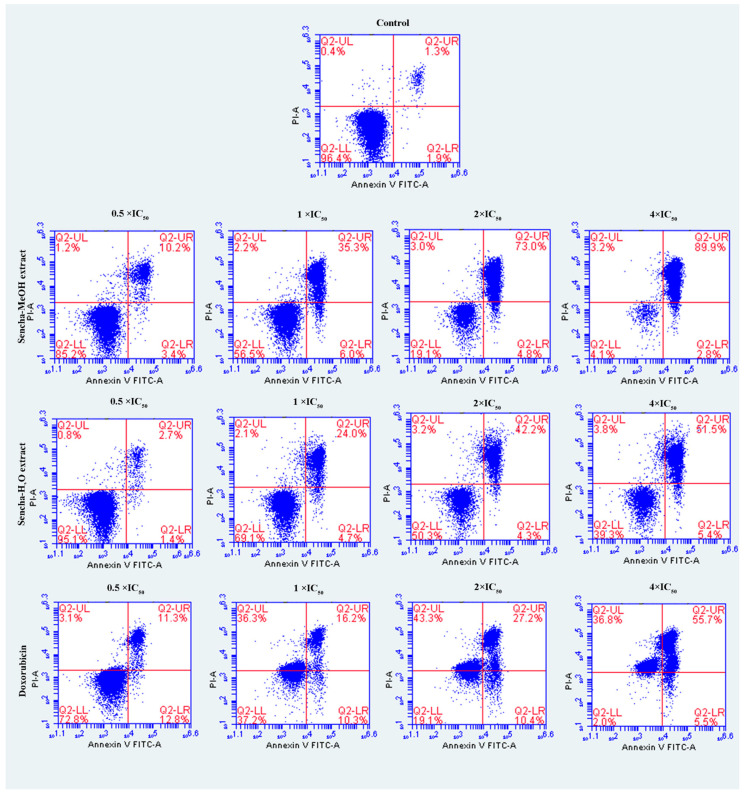
Assessment of apoptosis induced by sencha-MeOH extract, sencha-H_2_O extract, and doxorubicin on CCRF/CEM leukemia cells after 48 h as determined by annexin V/PI assay. Apoptosis was assessed by flow cytometry after annexin V-PI double staining. IC_50_ values were 8.38 μg/mL for sencha-MeOH extract, 11.50 μg/mL for sencha-H_2_O extract, 0.0093 μM for doxorubicin on CCRF/CEM cells. Necrotic cells lose membrane integrity, allowing PI entry. Q2-LL: viable cells exhibit annexin V-/PI-; Q2-LR: early apoptotic cells exhibit annexin V+/PI-; Q2-UR and Q2-UL: late apoptotic cells or necrotic cells exhibit annexin V+/PI+.

**Figure 6:**
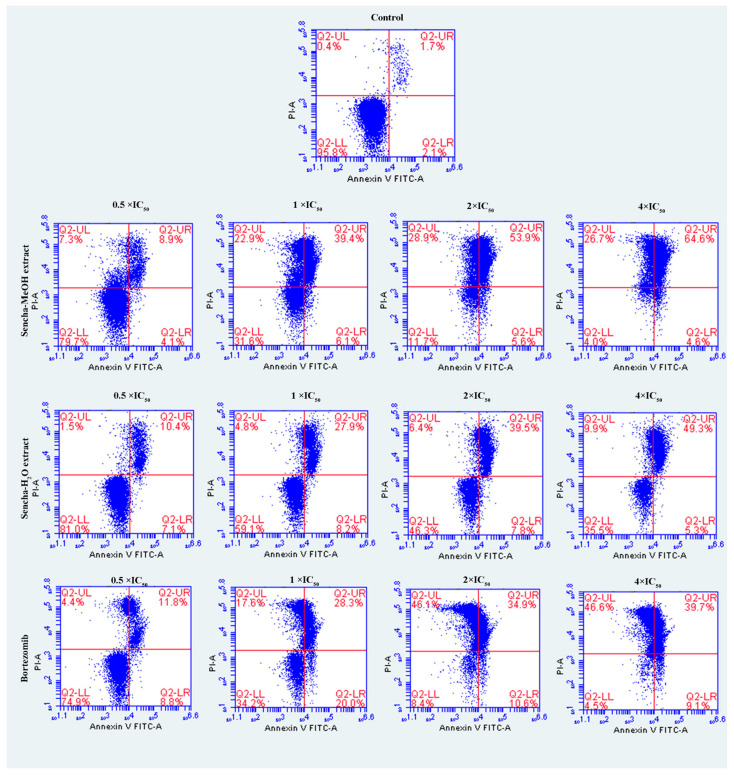
Assessment of apoptosis induced by sencha-MeOH extract, sencha-H_2_O extract, and bortezomib on KMS-12-BM cells after 48 h as determined by annexin V/PI assay. Apoptosis was assessed by flow cytometry after annexin V-PI double staining. IC_50_ values were 11.37 μg/mL for sencha-MeOH extract, 14.85 μg/mL for sencha-H_2_O extract, 0.0019 μM for bortezomib on KMS-12-BM cells. Necrotic cells lose membrane integrity, allowing PI entry. Q2-LL: viable cells exhibit annexin V-/PI-; Q2-LR: early apoptotic cells exhibit annexin V+/PI-; Q2-UR and Q2-UL: late apoptotic cells or necrotic cells exhibit annexin V+/PI+.

The cells were further investigated for the detection of early apoptosis, late apoptosis, and necrosis by annexin V/PI staining. Since the apoptosis was not obvious for 24-h exposure of sencha extract, we turned to monitored apoptosis after 48-h treatment. Sencha MeOH extract induced apoptosis on both CCRF/CEM and KMS-12-BM cells. At 4 × IC_50_ for example, sencha-MeOH extract induced 89.9% of late apoptosis/necrosis (annexin V+/PI +) cells and 2.8% of early apoptosis (annexin V+/PI-) in CCRF/CEM, respectively; sencha-H_2_O extract induced 51.5% of late apoptosis/necrosis and 5.4% of early apoptosis, respectively (Figure 5). In terms of KMS-12-BM cells, it presented 64.4% of late apoptosis/necrosis and 4.6% of early apoptosis towards sencha-MeOH extract at 4 × IC_50_, 49.3% of late apoptosis/necrosis and 5.3% of early apoptosis towards sencha- H_2_O extract (Figure 6). The percentages of late apoptosis/necrosis were 10.2%, 35.3%, 73.0%, and 89.9% for CCRF/CEM towards different concentrations of sencha-MeOH. Similarly, the proportion of late apoptosis/necrosis were 8.9%, 39.4%, 53.9%, and 64.6% for KMS-12-BM cells against sencha-MeOH extract. These findings represented an increase in apoptosis in a dose-dependent manner. Sencha-MeOH extract induced a higher extent of apoptosis versus sencha-H_2_O extract.

### 3.5. Measurements of ROS

**Figure 7:**
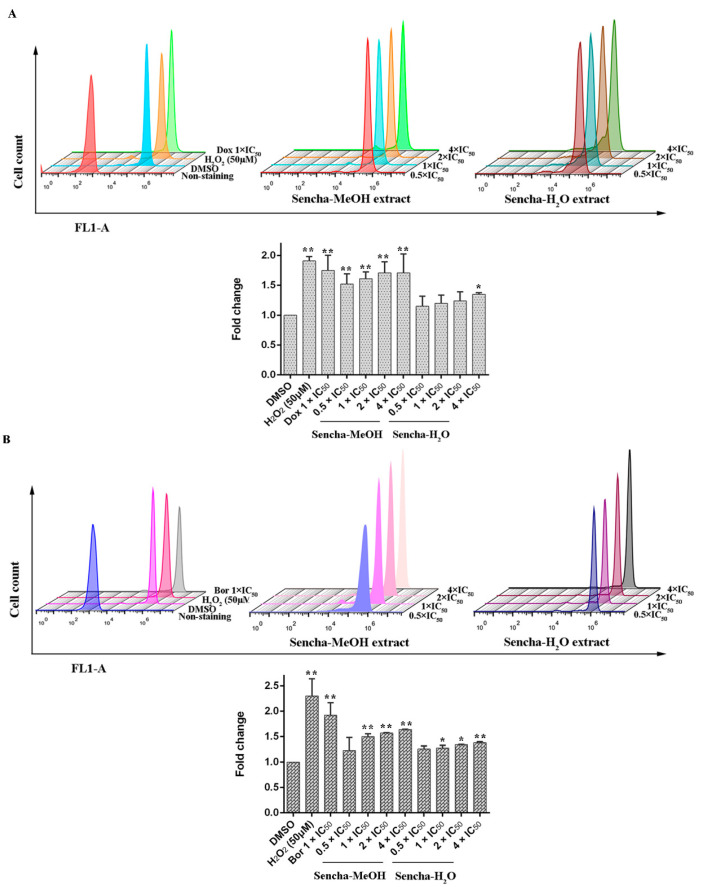
Induction of ROS level in CCRF/CEM (**A**) and KMS-12-BM (**B**) upon 48 h treatment with sencha-MeOH extract and sencha-H_2_O extract at a set of concentrations. IC_50_ values were 8.38 μg/mL for sencha-MeOH extract, 11.50 μg/mL for sencha-H_2_O extract, and 0.0093 μM for doxorubicin towards CCRF/CEM cells. IC_50_ values were 11.37 μg/mL for sencha-MeOH extract, 14.85 μg/mL for sencha-H_2_O extract, and 0.0019 μM for bortezomib towards KMS-12-BM cells. DMSO was used as the negative control, H_2_O_2_ (50 μM), doxorubicin (1-fold IC_50_), and bortezomib (1-fold IC_50_) as positive controls. Asterisks above bars denote *p*-values for one-way ANOVA analysis: * *p* < 0.05, ** *p* < 0.01 compared to DMSO control cells). Mean values ± SD were derived from three independent experiments.

Green tea polyphenols harbor both antioxidant and pro-oxidant effects polyphenols in cancer prevention: they can be direct antioxidants by scavenging ROS or be potent pro-oxidants by the formation of ROS to induce apoptosis [17,23,24,69]. Inhibition of cancer cell viability and induction of apoptosis by green tea polyphenols (ECG, EGCG, and EGC) in vitro have been reported to result from the generation of hydrogen peroxide and superoxide anion [69]. To determine the effects of the sencha extracts on the production of intracellular ROS levels, we used H2DCFDA staining. Comparing with DMSO controls, both positive controls, H_2_O_2_ and doxorubicin, could induce ROS levels at 1.91- and 1.75-fold (*p* < 0.01). Sencha-MeOH extracts could significantly stimulate ROS production in a dose-dependent manner in comparison with DMSO-treated control CCRF/CEM cells (*p* < 0.01), whereas sencha-H_2_O extract up to the concentration of 4-fold IC_50_ could induce ROS significantly (1.35-fold, Figure 7A). Similarly, sencha-MeOH extracts caused the increase of ROS production on KMS-12-BM cells with 1.23-, 1.50-, 1.57-, 1.64-fold respectively, with a higher degree than sencha-H_2_O extract (Figure 7B). Excessive ROS generations are known to evoke DNA damage [70,71]. These results demonstrated that oxidative stress generated by sencha extracts could be a reason for the apoptosis of CCRF/CEM and KMS-12-BM cells.

### 3.6. Assessment of MMP

**Figure 8:**
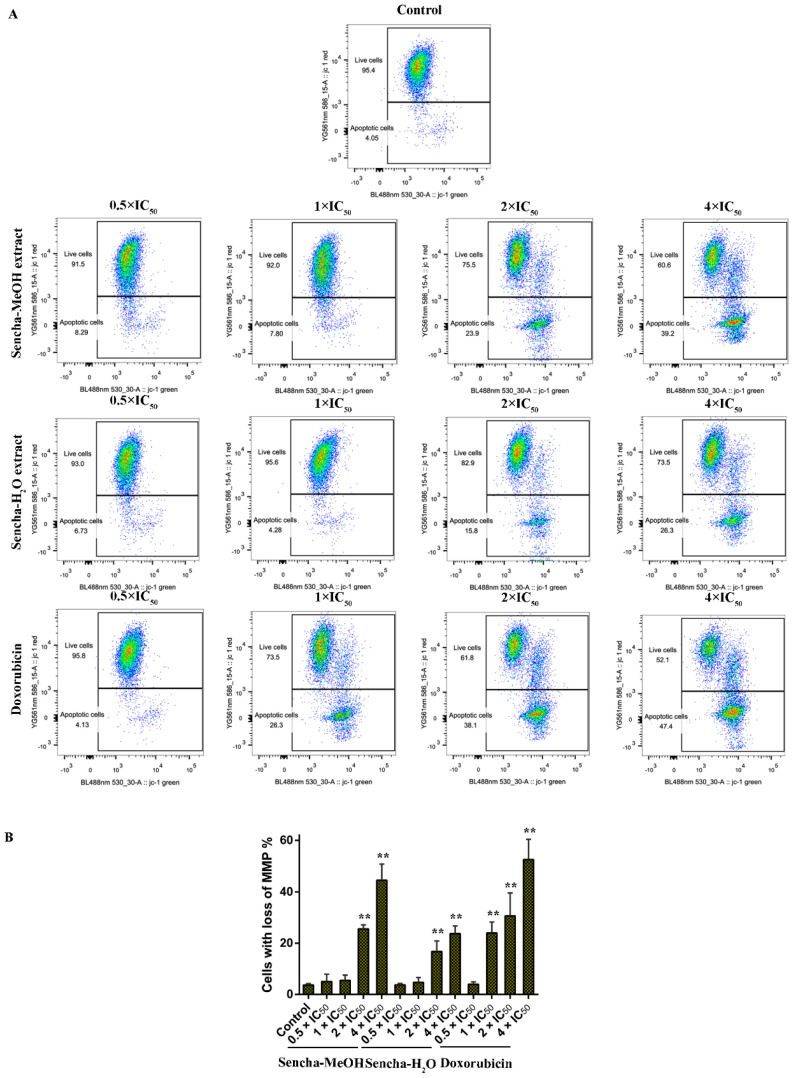
Representative images of JC-1 fluorescence with flow cytometry and statistical analysis of mitochondrial membrane potential on CCRF/CEM cells. (**A**) Cells were treated with DMSO as control and 0.5-, 1-, 2-, and 4-fold IC_50_ of sencha-MeOH/H_2_O extract and doxorubicin respectively for 24 h. (**B**) Statistical results of the apoptotic cell were defined as MMP collapse after 24 h treatment. IC_50_ values were 8.38 μg/mL for sencha-MeOH extract, 11.50 μg/mL for sencha-H_2_O extract, and 0.0093 μM for doxorubicin towards CCRF/CEM cells. Asterisks above bars denote *p*-values for one-way ANOVA analysis: ** *p* < 0.01 compared to DMSO control cells. Mean values ± SD were derived from three independent experiments.

**Figure 9:**
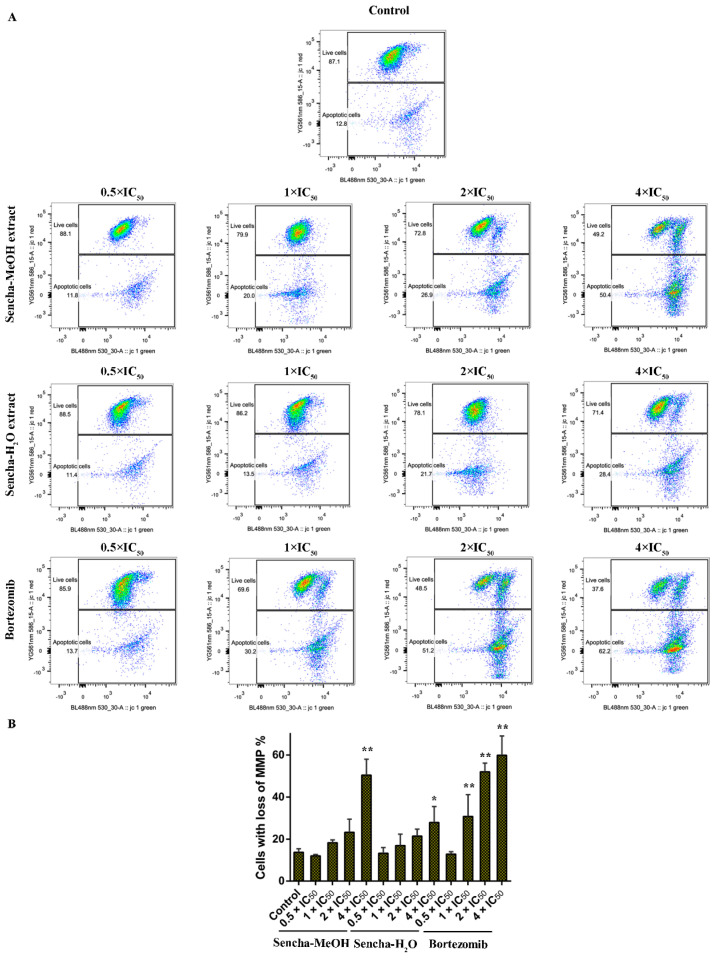
Representative images of JC-1 fluorescence with flow cytometry and statistical analysis of mitochondrial membrane potential on KMS-12-BM cells. (**A**) Cells were treated with DMSO as control and 0.5-, 1-, 2-, and 4-fold IC_50_ of sencha-MeOH/H_2_O extract and bortezomib respectively for 24 h. (**B**) Statistical results of the apoptotic cell were defined as MMP collapse after 24 h treatment. IC_50_ values were 11.37 μg/mL for sencha-MeOH extract, 14.85 μg/mL for sencha-H_2_O extract, and 0.0019 μM for bortezomib towards KMS-12-BM cells. Asterisks above bars denote *p*-values for one-way ANOVA analysis: * *p* < 0.05, ** *p* < 0.01 compared to DMSO control cells. Mean values ± SD were derived from three independent experiments.

Mitochondria are the major site of ROS production. Increased intracellular ROS in mitochondria causes oxidative stress, loss of MMP, DNA damage, and finally the death of cancer cells [70,71,72]. MMP alterations can indicate mitochondrial function. The mitochondrial dysfunction was observed on human gastric cancer after the treatment of tea catechin EGCG [73]. Since we found that both sencha-MeOH and sencha-H_2_O extracts could increase ROS generation of CCRF/CEM and KMS-12-BM cells, we further measured MMP alterations on both cells after 24h exposure. CCRF/CEM cells stained with the MMP-specific dye JC-1 revealed a sharp shift from red (unaltered potential) to green fluorescence (impaired potential) following treatment with 2- and 4-fold IC_50_ of sencha-MeOH/-H_2_O extracts (Figure 8); the percentages of KMS-12-BM cells with MMP collapse were up to 50.4% ± 7.60% and 27.9% ± 2.96% towards 4-fold IC_50_ of sencha-MeOH/-H_2_O extracts respectively (Figure 9). Hence our data suggested that sencha tea caused mitochondrial dysfunction of CCRF/CEM and KMS-12-BM cells. 

### 3.7. COMPARE and Hierarchical Cluster Analyses of Microarray Data

It is well documented that the response of cancer cells to phytochemicals is determined by multiple factors and complex interactions [74]. To identify novel putative factors associated with cellular response to main tea catechins (EGCG and EGC), we applied COMPARE analysis. No microarray data were recorded about EGCG, so we turned to focus on EGC based on the Novartis microarray platform and correlated the transcriptome-wide RNA expression of the NCI 60 cell lines by COMPARE analyses with the log_10_IC_50_ values for EGC by Pearson’s rank correlation coefficients. The top 20 genes with direct and the top 20 genes with inverse correlation coefficients for EGC were listed in Table 3. These gene-encoding proteins belonged to diverse functional groups, such as cell morphology (*APOE*, *CACNA1D*, *CDK5R1*, *NDEL1*, *PTPRZ1*), cellular growth and proliferation (*RACK1*, *SLC25A2*, *TINM8A*), cell cycle (*APOE*, *BUB3*, *CDH1*, *CDK5R1*, *RACK1*, *RAN*, *RFPL1/RFPL3*, *TCERG1*), cell death and survival (*ATG12*, *ATP2A2*, *TCERG1*, *SLC25A5*, *RACK1, HLF*).

**Figure 10:**
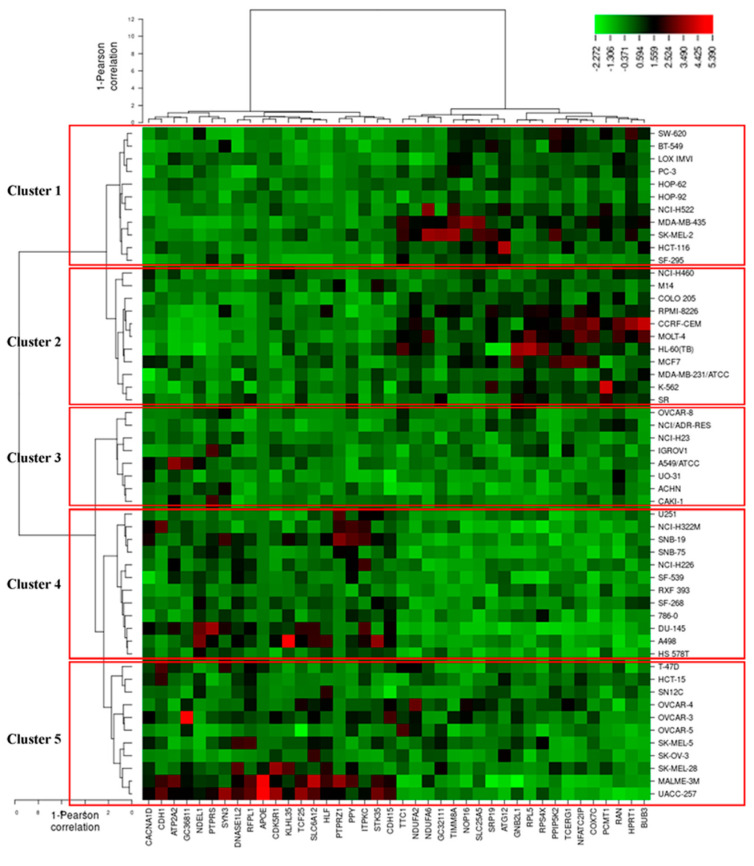
Heat-map obtained by hierarchical cluster analysis of transcriptome-wide expression profiling of 53 NCI tumor cell lines correlating to sensitivity and resistance towards EGC.

To predict whether these 40 genes identified by the COMPARE algorithm involved in sensitivity or resistance of the cells to EGC, they were then subjected to hierarchical cluster analyses. The mRNA expression data of these cell lines but not the log_10_IC_50_ values of 53 human tumor cell lines towards EGC were included. The resulting dendrogram with the 53 cell lines could be divided into five clusters, as shown in Figure 10. We subsequently distributed these cell lines into sensitive or resistant cells towards EGC. The median log_10_IC_50_ value (−4.7 M) was taken as a cut-off to differentiate between sensitive or resistant cells towards EGC. Cluster 1 and 2 contained cell lines that were in its majority sensitive to EGC, whereas cluster 3, 4, and 5 contained in its majority resistant ones. Based on the chi-square test, the distribution of sensitive or resistant cell lines was statistically significant (*p =* 0.037, Table 4). This result indicates that the mRNA expression of these genes causes a dendrogram branching that was able to predict the responsiveness of cell lines to EGC.

Tumors frequently confer resistance to a broad spectrum of anti-cancer drugs via mutational and network-level mechanisms [75,76]. In this study, 7 genes among the selected 40 genes have been documented to correlate with drug resistance. *SPTPRS* is known to develop adaptive resistance to MEK/ERK inhibitors through SRC activation [77]. Novel fusion genes *NDEL1-PDGFRB* and *PTPRZ1–MET* have been recently reported to associate with chemoresistance resistance [78,79]. Epigenetic loss of *CDH1* correlates with doxorubicin-induced multidrug resistance in human hepatocellular carcinoma cells [80]. *PAK4*, which is essential for Ras-induced cell cycle progression, confers cisplatin resistance in gastric cancer cells via PI3K/Akt- and MEK/ERK-dependent pathways [81]. *HPRT1* activity loss is associated with resistance to thiopurine in ALL [82]. *RPS4X*, which is involved in cellular translation and proliferation, results in consistent resistance to cisplatin in ovarian cancer [83]. The other genes have not reported in terms of their relation to drug resistance yet. Together, the 7 genes may be the determinants of cellular resistance towards EGC.

### 3.8. Ingenuity Pathway Analysis (IPA)

**Figure 11:**
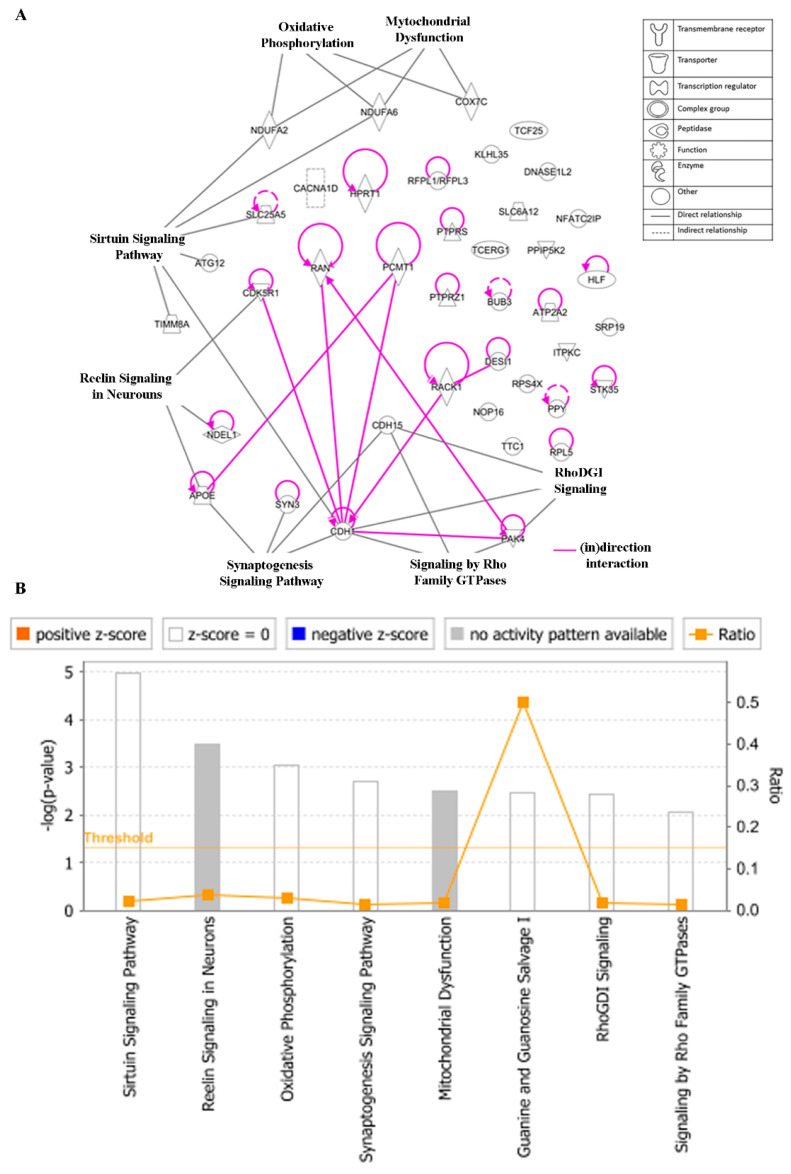
Canonical pathways (**A**) and the corresponding –log (*p*-value) (**B**) for genes determining cellular responsiveness towards EGC with IPA. Significance *p*-values were calculated based on Fisher’s right-tailed exact test. The –log (*p*-value) is shown on the *y*-axis of the bar chart. The color of the bars indicates the activity (orange bars) or the inhibition (blue bars) of the predicted pathways. In this analysis, only significant results were shown. By default, IPA applies a –log (*p*-value) cutoff of 1.3 (threshold). Pathways with a *p*-value equal to or greater than (less significant than) 0.05 are not shown. The orange and blue colored bars indicate predicted pathway activation or inhibition (z-score). White bars are those with a z-score at or very close to 0. Gray bars indicate pathways with no prediction currently available.

To gain insight into the networks and possible functional pathways, the 40 regulated genes determining cellular responsiveness to EGC were subjected to IPA. Firstly, we analyzed the key canonical pathways. The identified pathways belonged to signal transduction (signaling by the Rho Family GTPase and RhoDGI signaling), neurological pathways (Reelin signaling in Neurons, synaptogenesis signaling pathway), mitochondrial dysfunction, oxidative phosphorylation, and sirtuin signaling pathway (Figure 11A). The significance of these pathways is presented as –log (*p*-value) in Figure 11B.

**Figure 12:**
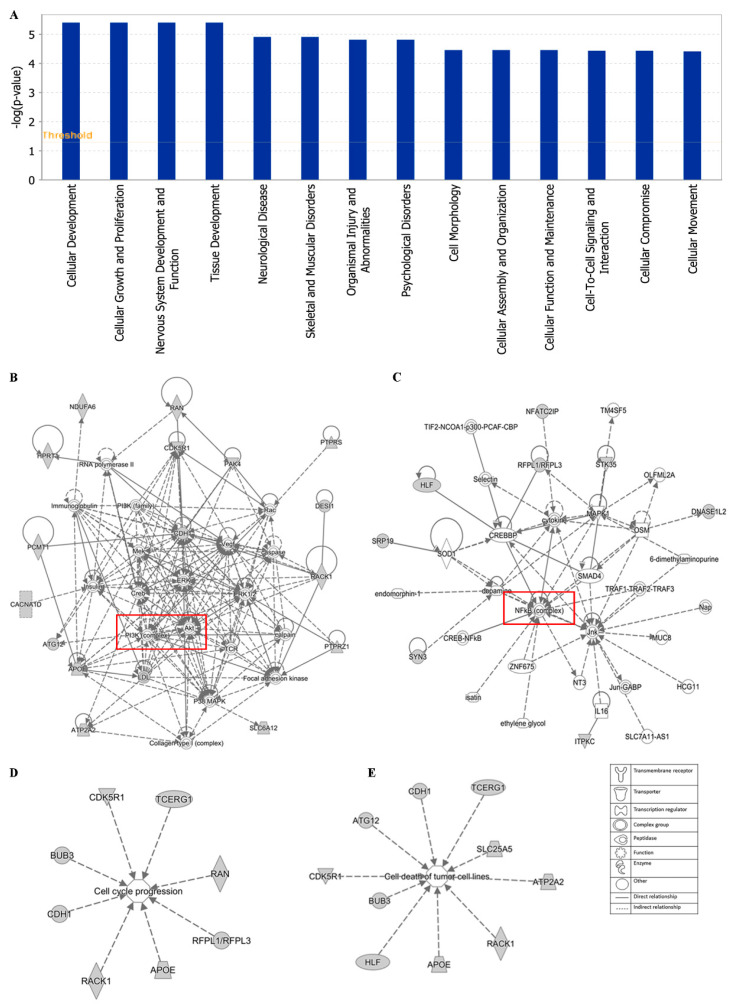
Statistically significant biological function analysis (**A**), PI3K/Akt network (**B**), NF-κB network (**C**), set of 8 genes involved in the cell cycle (**D**) and set of 10 genes involved in cell death (**E**) using IPA.

Additionally, cellular development, cellular growth and proliferation, cell morphology cellular assembly and organization were identified by IPA as biological functions (Figure 12A). IPA network analysis also highlighted the top two pathways, including the PI3K/Akt and NF-κB pathway, from our selected genes (Figure 12B,C). Networks of 8 genes and 10 genes were involved in cell cycle and cell death (apoptosis), respectively (Figure 12D,E).

The top network of EGC towards cancer depicted by IPA was PI3K/Akt pathway with 16 genes among the 40 selected genes involved in this pathway (Figure 12B). PI3K/Akt pathway is an important intracellular mediator. Akt acts downstream of PI3K to regulate many biological processes, including cell cycle progression, cell survival, apoptosis, and neoplastic transformation, through phosphorylation of numerous cellular proteins, such as NF-κB, Bad, and caspase-9 [84]. The link between the PI3K/Akt pathway and cancer makes this pathway an attractive target for therapeutic strategies. Although neither PI3K nor Akt were included in the 40 selected genes responding to EGC, PI3K/Akt pathway works with other signal transduction directly or indirectly, such as MEK/ERK pathway, VEGF/VEGFR signaling, adenosine monophosphate-activated protein kinase (MAPK), and caspases. Another major tea catechin, EGCG, the acid ester of EGC and GA, was also reported to possess antiangiogenic effects via inhibiting PI3K/Akt and MEK/ERK pathways synergistically [85]. EGCG promoted apoptosis in bladder cancer cells via modulation of the PI3K/Akt pathway and Bcl-2 family proteins [28]. These studies demonstrated that the PI3K/Akt pathway may play an important role in green tea catechins against cancer.

The second network of EGC towards cancer was the NF-κB pathway with 8 genes among the 40 selected genes involved in this pathway, including *DNASE1L2*, *ITPKC*, *SRP19*, *HLF*, *STK35*, *MFATC2IP*, *SYN3*, and *RFPL1/RFPL3* (Figure 12C). NF-κB, a sequence-specific transcription factor, which can be activated by proinflammatory cytokines, free radicals, and DNA damage, has been increasingly recognized as a crucial player in many steps of cancer initiation and progression, metastasis [86,87]. Overexpression of NF-κB has been observed on diverse types of solid cancer as well as hematologic neoplasias [88,89]. Suppression of NF-kB in myeloid cells or tumor cells usually leads to tumor regression, which makes the NF-kB pathway a promising therapeutic target. The high binding affinity of tea catechins EGC, EGCG, and ECG with NF-κB have been reported, implying the potential inhibition of tea catechins on NF-κB [90]. Both green tea extract and the tea catechins could decrease the expression of NF-κB, accompanied by the downregulation of matrix metalloproteinase (MMP)-9 and -3 [91,92]. Thus, the NF-κB pathway contributes to the inhibition of cancer by green tea.

Therefore, these results draw a diverse picture of different gene functions and cancer-associated mechanisms, implying that sencha tea exerts multifactorial determined cytotoxicity towards cancer cells, as most natural products do.

## 4. Conclusions and Perspectives

In our investigation, GNPS- and Cytospace-based chemical profiles of sencha tea may explain its effects against cancer in terms of ROS induction, G0/G1 or G2M cell cycle arrest, MMP decline, and apoptosis induction in vitro. Moreover, the exploratory and combined tool of microarray, COMPARE, and IPA analyses propose that various functional groups of genes, especially those involved in the regulation of the PI3K/Akt and NF-κB networks may contribute to the anti-cancer property of sencha tea. Collectively, these outcomes support the chemopreventive property of sencha tea against hematologic malignances through multiple routes of action in vitro.

However, it deserves further attention to disclose the full range of molecular mechanisms that determine the responsiveness of cancer cells to sencha tea. Particularly, the role of intracellular or extracellular ROS on cancer towards sencha tea needs more intensive and in vivo researches to validate. Still, sencha-MeOH extract showed a stronger effect than sencha-H_2_O extract based on our results, which indicated that higher tea catechins could gain more health benefits. Whereas the accurate dose of tea catechins has not been decided yet. Dose-related differences in the effects of green tea or tea catechins in cancer applications needs further clarification.

## Figures and Tables

**Table 1:** IC_50_ value of sencha extracts towards CCRF/CEM and CEM/ADR5000 cell lines

Cell Lines	Sencha-MeOH		Sencha-70% MeOH		Sencha-H_2_O		Doxorubicin	
	IC50(μg)	Resistance Degree	IC50(μg)	Resistance Degree	IC50(μg)	Resistance Degree	IC50(μM)	Resistance Degree
CCRF/CEM	8.38 ± 0.72	2.21	11.34 ± 1.86	1.90	11.50 ± 1.30	2.94	0.0093 ± 0.00	7758.78
CEM/ADR5000	18.52 ± 1.98		21.57 ± 2.69		33.8 ± 3.55		72.43 ± 2.61	

The data are shown as mean values SD of three independent experiments with every six parallel measurements. Degrees of resistance was calculated by dividing the IC_50_ value of the CEM/ADR5000 over the IC_50_ value of sensitive CCRF/CEM cells.

**Table 2:** IC_50_ value of sencha extracts and bortezomib towards MM cell lines

Cell Line	KMS-12-BM	MolP-8	KMS-11	NCI-H929	AMO-1	L-363	OPM-2	JJN-3	RPMI-8226
Sencha-MeOH extract (μg)	11.37 ± 1.03	16.51 ± 2.32	17.13 ± 2.53	18.23 ± 0.59	39.26 ± 2.72	61.65 ± 9.37	42.20 ± 4.80	51.74 ± 3.74	68.90 ± 6.25
Sencha-H_2_O extract (μg)	14.85 ± 1.44	24.38 ± 3.58	52.66 ± 4.22	25.52 ± 5.56	42.58 ± 2.81	71.19 ± 1.88	76.63 ± 1.36	>80	>80
Bortezomib (μM)	0.0019 ± 0.00013	0.0018 ± 0.000078	0.0016 ±0.00038	0.0014 ± 0.00032	0.0020 ± 0.000024	0.0018± 0.00016	0.0018 ± 0.00020	0.0015 ± 0.00044	0.0016 ± 0.00035

The data are shown as mean values SD of three independent experiments with every six parallel measurements.

**Table 3:** Correlation coefficients of mRNA expression to log_10_IC_50_ values obtained using COMPARE analyses for 53 NCI cancer cell lines and gene function obtained from gene cards and the UniProt database

COMPARE	Experimental	GenBank	Gene		
Coefficient	ID	Accession	Symbol	Name	Function
−0.605	GC39119	Y11999	*ITPKC*	Inositol 1,4,5-trisphosphate 3-kinase C RNA	Phosphorylation of inositol 2,4,5-triphosphate to inositol 2,4,5,6-tetraphosphate
−0.596	GC35270	U40317	*PTPRS*	Protein tyrosine phosphatase, receptor type, S RNA	Inhibition of neurite and axonal outgrowth
−0.527	GC37416	AF038203	*NDEL1*	Nude nuclear distribution gene E homolog (A. nidulans)-like 1 RNA	Organization of the cellular microtubule array and microtubule anchoring at the centrosome
−0.526	GC32502	M12529	*APOE*	Apolipoprotein E RNA	Function in lipoprotein-mediated lipid transport
−0.523	GC31111	U27699	*SLC6A12*	Solute carrier family 6 (neurotransmitter transporter, betaine/GABA), member 12 RNA	Regulation of gabaergic transmission in the brain
−0.516	GC34680	U62647	*DNASE1L2*	Deoxyribonuclease I-like 2 RNA	Breakdown of the nucleus during corneocyte formation of epidermal keratinocytes
−0.512	GC32691	X80343	*CDK5R1*	Cyclin-dependent kinase 5, regulatory subunit 1 (p35) RNA	Required for neurite outgrowth and cortical lamination
−0.511	GC29695	M76558	*CACNA1D*	Calcium channel, voltage-dependent, L type, alpha 1D subunit RNA	Mediate the entry of calcium ions into excitable cells
−0.511	GC34733	W27128	*STK35*	Unknown RNA	Association with PDLIM1 is controversial
−0.509	GC30627	X68985	*HLF*	Hepatic leukemia factor RNA	Accumulate according to a robust circadian rhythm
−0.503	GC30214	M23115	*ATP2A2*	Atpase, Ca++ transporting, cardiac muscle, slow twitch 2 RNA	Catalyze the hydrolysis of ATP coupled with the translocation of calcium
−0.495	GC30715	AA471042	*KLHL35*	Kelch-like 35 (Drosophila) RNA	Interactions with 2 proteins
−0.494	GC37908	AF046873	*SYN3*	Synapsin III RNA	Involved in the regulation of neurotransmitter release and synaptogenesis
−0.494	GC33357	M93426	*PTPRZ1*	Protein tyrosine phosphatase, receptor-type, Z polypeptide 1 RNA	Negatively regulate oligodendrocyte precursor proliferation in the embryonic spinal cord
−0.489	GC35321	AA844998	*PPY*	Pancreatic polypeptide RNA	A regulator of pancreatic and gastrointestinal functions
−0.488	GC34918	AJ010228	*RFPL1*	Ret finger protein-like 1 RNA	Negatively regulate the G2-M phase transition by promoting cyclin B1/CCNB1 and CDK1 proteasomal degradation
−0.475	GC34162	L08599	*CDH1*	Cadherin 1, type 1, E-cadherin (epithelial) RNA	Contribute to the sorting of heterogeneous cell types
−0.473	GC36409	AI332820	*TCF25*	Transcription factor 25 (basic helix-loop-helix) RNA	Control of cell death and repress transcription of SRF
−0.473	GC33285	D83542	*CDH15*	Cadherin 15, type 1, M-cadherin (myotubule) RNA	Contribute to the sorting of heterogeneous cell types
−0.47	GC36811	AF005046	*PAK4*	P21 protein (Cdc42/Rac)-activated kinase 4 RNA	Serve as targets for the small GTP binding proteins Cdc42 and Rac
0.501	GC28045	M31642	*HPRT1*	Hypoxanthine phosphoribosyltransferase 1 RNA	Play a central role in the generation of purine nucleotides
0.474	GC29847	AF017789	*TCERG1*	Transcription elongation regulator 1 RNA	Regulate transcription elongation in a TATA box-dependent manner
0.456	GC35203	AA152202	*NFATC2IP*	Nuclear factor of activated T-cells, cytoplasmic, calcineurin-dependent 2 interacting protein RNA	Regulate the magnitude of NFAT-driven transcription of a specific subset of cytokine genes
0.452	GC27723	U46570	*TTC1*	Tetratricopeptide repeat domain 1 RNA	Unfolded protein binding
0.45	GC37383	AI708889	*COX7C*	Cytochrome c oxidase subunit viic RNA	Cytochrome-c oxidase activity
0.445	GC36655	U14966	*RPL5*	Ribosomal protein L5 RNA	Responsible for the synthesis of proteins
0.438	GC38242	X12791	*SRP19*	Signal recognition particle 19kda RNA	Bind directly to 7S RNA and mediates binding of the 54 kda subunit of the SRP
0.436	GC31697	AI553745	*NOP16*	NOP16 nucleolar protein homolog (yeast) RNA	RNA binding
0.436	GC28146	J02683	*SLC25A5*	Solute carrier family 25 (mitochondrial carrier; adenine nucleotide translocator), member 5 RNA	Catalyze the exchange of cytoplasmic ADP with mitochondrial ATP across the mitochondrial inner membrane
0.434	GC29122	AF054183	*RAN*	RAN, member RAS oncogene family RNA	Participate both to the import and the export from the nucleus of proteins and RNAs
0.421	GC37648	M58458	*RPS4X*	Ribosomal protein S4, X-linked RNA	Positive regulation of cell population proliferation
0.421	GC28143	D25547	*PCMT1*	Protein-L-isoaspartate (D-aspartate) O-methyltransferase RNA	Repair and/or degradation of damaged proteins
0.418	GC30306	U66035	*TIMM8A*	Translocase of inner mitochondrial membrane 8 homolog A (yeast) RNA	A chaperone-like protein that protects the hydrophobic precursors from aggregation
0.414	GC30977	AF047185	*NDUFA2*	Unknown RNA	Complex I functions in the transfer of electrons from NADH to the respiratory chain
0.414	GC35707	AA151922	*ATG12*	ATG12 autophagy related 12 homolog (*S. Cerevisiae*) RNA	Involved in autophagy vesicles formation
0.411	GC29399	AI223047	*NDUFA6*	NADH dehydrogenase (ubiquinone) 1 alpha subcomplex, 6, 14kDa RNA	Complex I functions in the transfer of electrons from NADH to the respiratory chain
0.408	GC38177	AB007893	*PPIP5K2*	Diphosphoinositol pentakisphosphate kinase 2 RNA	Act in concert with the IP6K kinases IP6K1, IP6K2, and IP6K3 to synthesize the diphosphate group-containing inositol pyrophosphates diphosphoinositol pentakisphosphate
0.401	GC37613	M24194	*GNB2L1*	Guanine nucleotide binding protein (G protein), beta polypeptide 2-like 1 RNA	Unknown
0.399	GC37789	AF047473	*BUB3*	Budding uninhibited by benzimidazoles 3 homolog (yeast) RNA	Regulate chromosome segregation during oocyte meiosis.
0.398	GC32111	R38263	*DESL1*	PPPDE peptidase domain containing 2 RNA	Deconjugate SUMO1, SUMO2, and SUMO3 from some substrate proteins

**Table 4:** Clusters of NCI 53 tumor cell lines gained by hierarchical cluster analyses for EGC

	Sensitive	Resistant
Partition (log_10_ IC_50_)	<−4.7 M	≥−4.7 M
Cluster 1	8	3
Cluster 2	8	3
Cluster 3	2	6
Cluster 4	3	9
Cluster 5	4	7
Chi-square test	*p* = 0.037	

The median log_10_IC_50_ value (−4.7 M) for EGC was used as a cut-off to classify tumor cell lines as ‘sensitive’ or ‘resistant’ cells.

## Supplementary Materials

The following are available online at https://www.mdpi.com/2218-273X/10/7/1000/s1, Figure S1: Distribution of cell cycle of CCRF/CEM upon 24 h treatment with sencha-MeOH extract, sencha-H2O extracts, doxorubicin at a set of concentrations, Figure S2: Distribution of cell cycle of CCRF/CEM upon 48 h treatment with sencha-MeOH extract, sencha-H2O extracts, doxorubicin at a set of concentrations, Figure S3: Distribution of cell cycle of CCRF/CEM upon 72 h treatment with sencha-MeOH extract, sencha-H2O extracts, doxorubicin at a set of concentrations, Figure S4: Distribution of cell cycle of KMS-12-BM upon 24 h treatment with sencha-MeOH extract, sencha-H2O extracts and bortezomib at a set of concentrations, Figure S5: Distribution of cell cycle of KMS-12-BM upon 48 h treatment with sencha-MeOH extract, sencha-H2O extracts and bortezomib at a set of concentrations, Figure S6: Distribution of cell cycle of KMS-12-BM upon 72 h treatment with sencha-MeOH extract, sencha-H2O extracts and bortezomib at a set of concentrations.
