# Supplementary material for: Chemopreventive Property of Sencha Tea Extracts towards Sensitive and Multidrug-Resistant Leukemia and Multiple Myeloma Cells
Source: Biomolecules. 2020 Jul 4;10(7):1000. doi: 10.3390/biom10071000 (PMC7407630; doi:10.3390/biom10071000)
Supplement: Supplementary file 1 [file biomolecules-10-01000-s001.pdf]

Chemopreventive property of Sencha tea extracts towards sensitive and multidrug-resistant leukemia and multiple myeloma cells

（Supplementary　Materials）

Xiaohua Lu [1], Mohamed E. M. Saeed [1], Mohamed-Elamir F. Hegazy [1,2], Christopher J. Kampf [3], and Thomas Efferth [1,*]

[1] Department of Pharmaceutical Biology, Institute of Pharmacy and Biochemistry, Johannes Gutenberg University, Staudinger Weg 5, 55128 Mainz, Germany; xiaohulu@uni-mainz.de (X.L.); saeedm@uni-mainz.de (M.E.M.S.); elamir77@live.com (M.E.F.H.)

[2] Chemistry of Medicinal Plants Department, National Research Centre, 33 El-Bohouth St., Dokki, Giza 12622, Egypt

[3] Department for Chemistry, Johannes Gutenberg University Mainz, Duesbergweg 10-14, 55128 Mainz, Germany; kampfc@uni-mainz.de

* Correspondence: efferth@uni-mainz.de; Tel.: +49-6131-392-5751; Fax: +49-6131-392-3752

1. Supplementary Figures (Figure S1-S6)

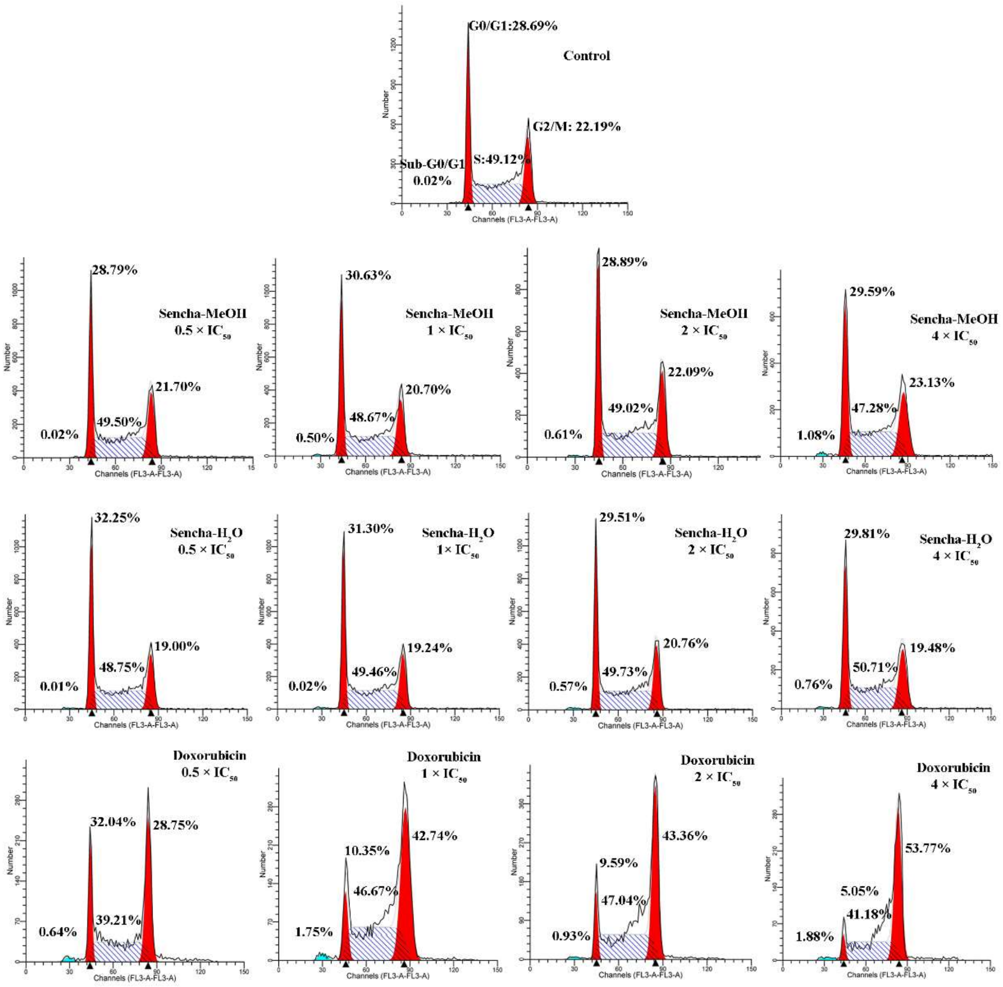

Figure S1. Distribution of cell cycle of CCRF/CEM upon 24 h treatment with sencha-MeOH extract, sencha-H_2O extracts, doxorubicin at a set of concentrations. IC_{50} values were 8.38 μg/mL for sencha-MeOH extract, 11.50 μg/mL for sencha-H_2O extract, 0.0093 μM for doxorubicin towards CCRF/CEM cells.

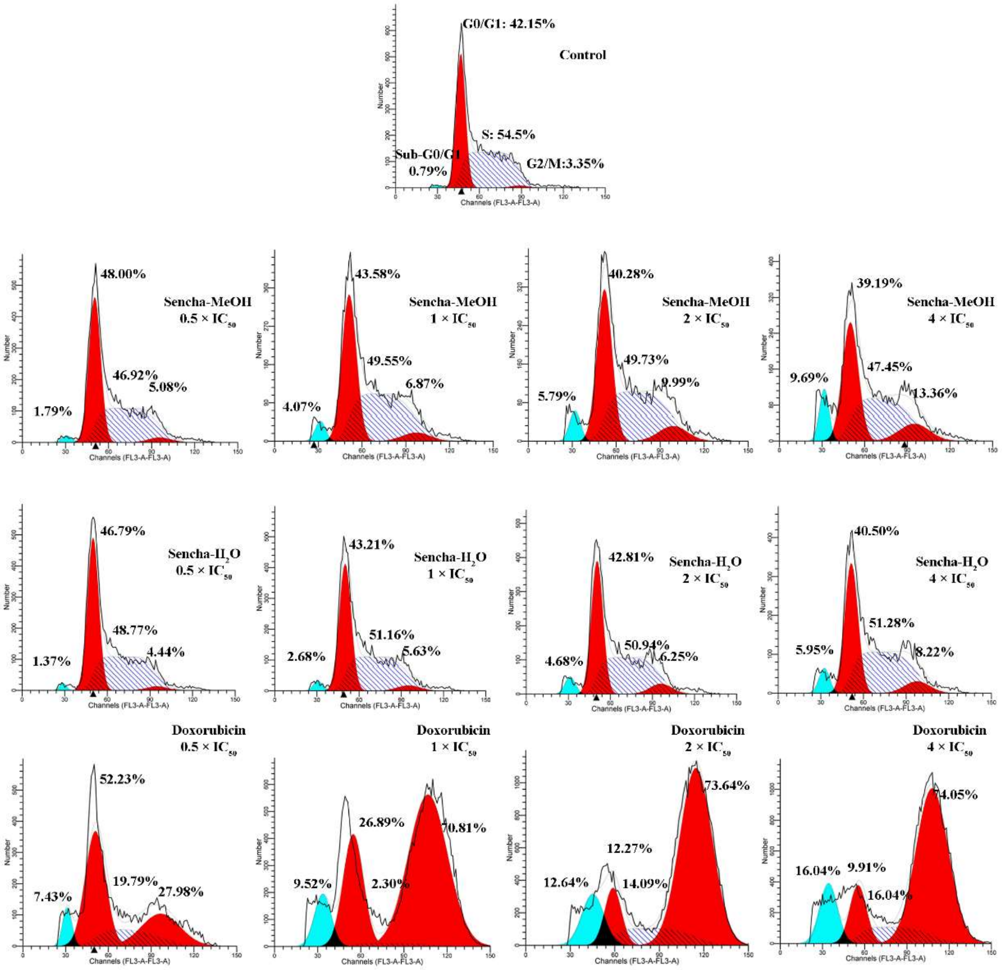

Figure S2. Distribution of cell cycle of CCRF/CEM upon 48 h treatment with sencha-MeOH extract, sencha-H_2O extracts, doxorubicin at a set of concentrations. IC_{50} values were 8.38 μg/mL for sencha-MeOH extract, 11.50 μg/mL for sencha-H_2O extract, 0.0093 μM for doxorubicin towards CCRF/CEM cells.

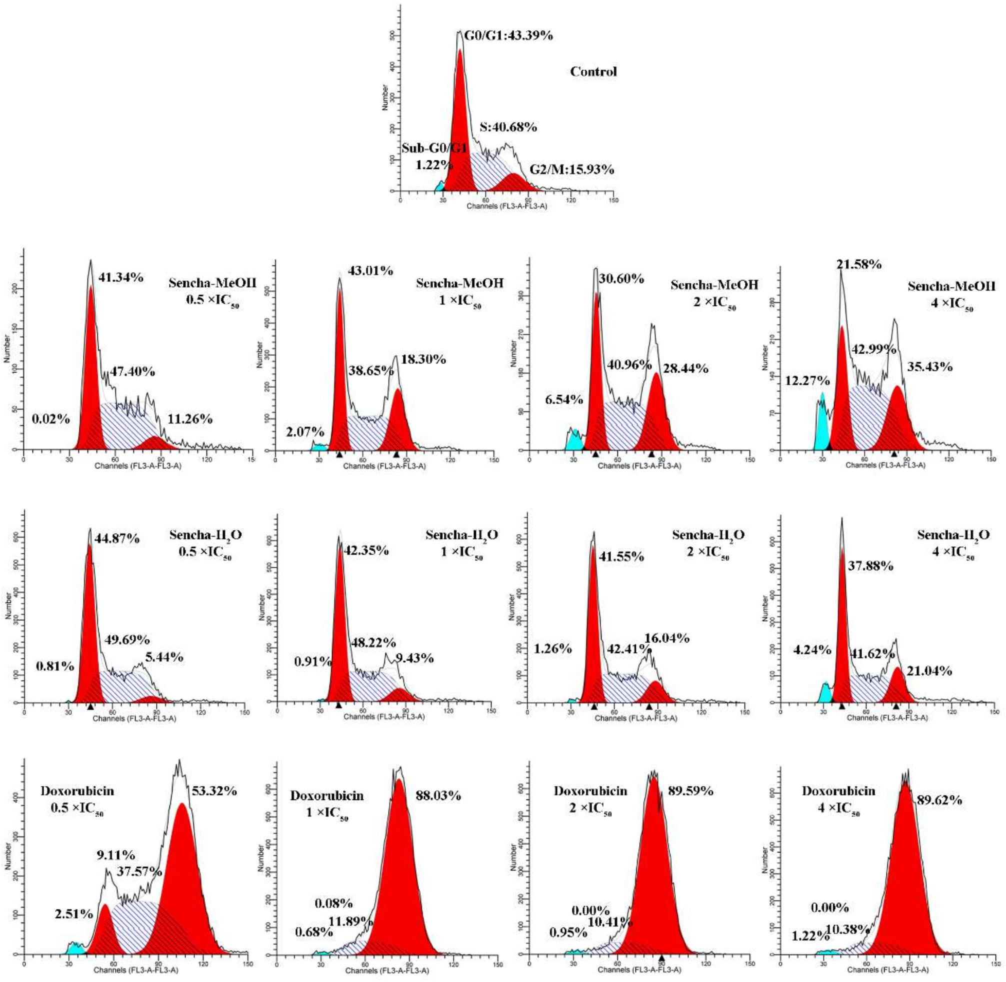

Figure S3. Distribution of cell cycle of CCRF/CEM upon 72 h treatment with sencha-MeOH extract, sencha-H_2O extracts, doxorubicin at a set of concentrations. IC_{50} values were 8.38 μg/mL for sencha-MeOH extract, 11.50 μg/mL for sencha-H_2O extract, 0.0093 μM for doxorubicin towards CCRF/CEM cells.

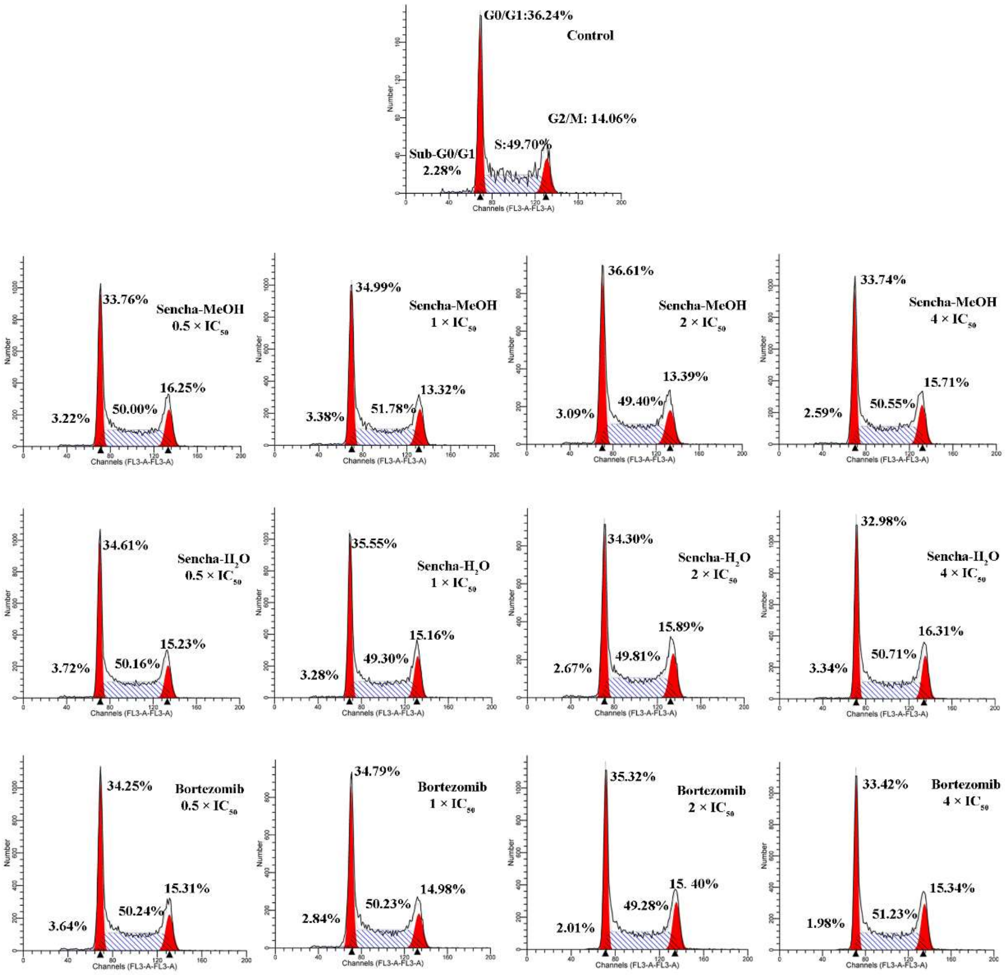

Figure S4. Distribution of cell cycle of KMS-12-BM upon 24 h treatment with sencha-MeOH extract, sencha-H_2O extracts and bortezomib at a set of concentrations. IC_{50} values were 11.37 μg/mL for sencha-MeOH extract, 14.85 μg/mL for sencha-H_2O extract, 0.0019 μM for bortezomib towards KMS-12-BM cells.

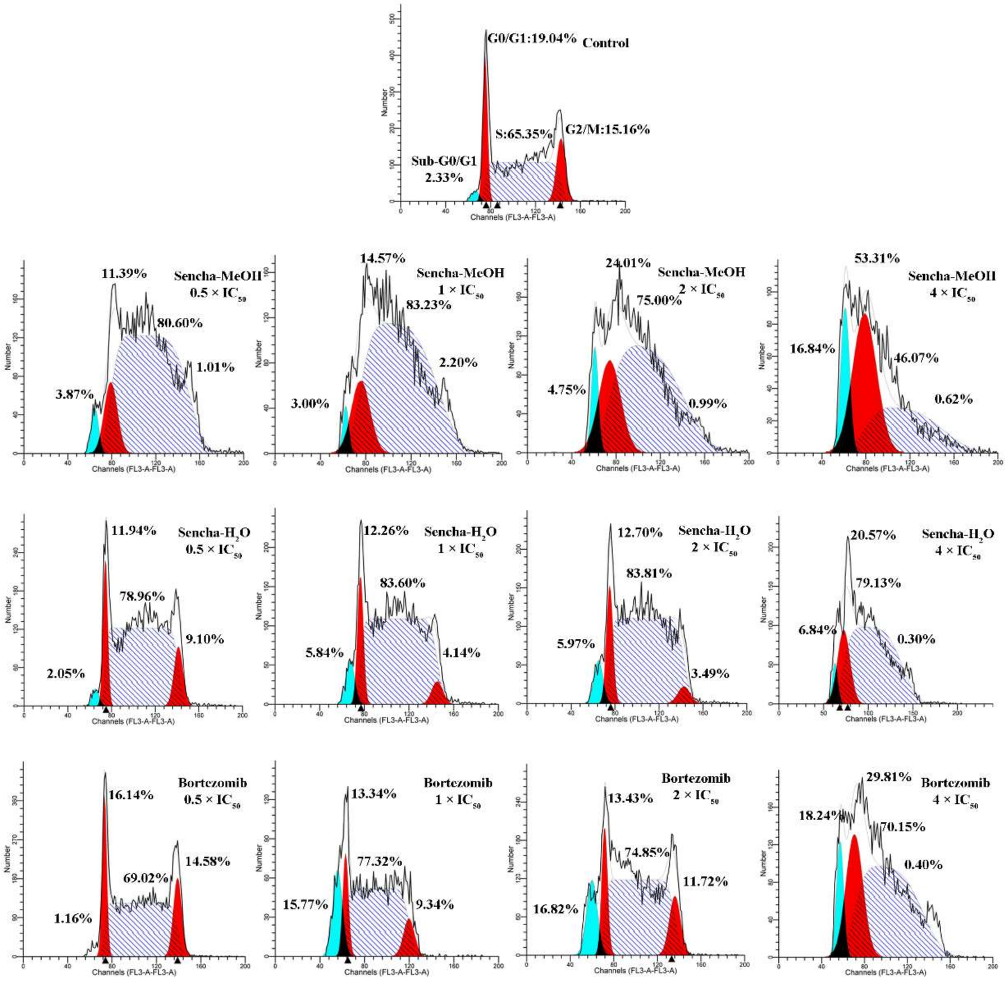

Figure S5. Distribution of cell cycle of KMS-12-BM upon 48 h treatment with sencha-MeOH extract, sencha-H_2O extracts and bortezomib at a set of concentrations. IC_{50} values were 11.37 μg/mL for sencha-MeOH extract, 14.85 μg/mL for sencha-H_2O extract, 0.0019 μM for bortezomib towards KMS-12-BM cells.

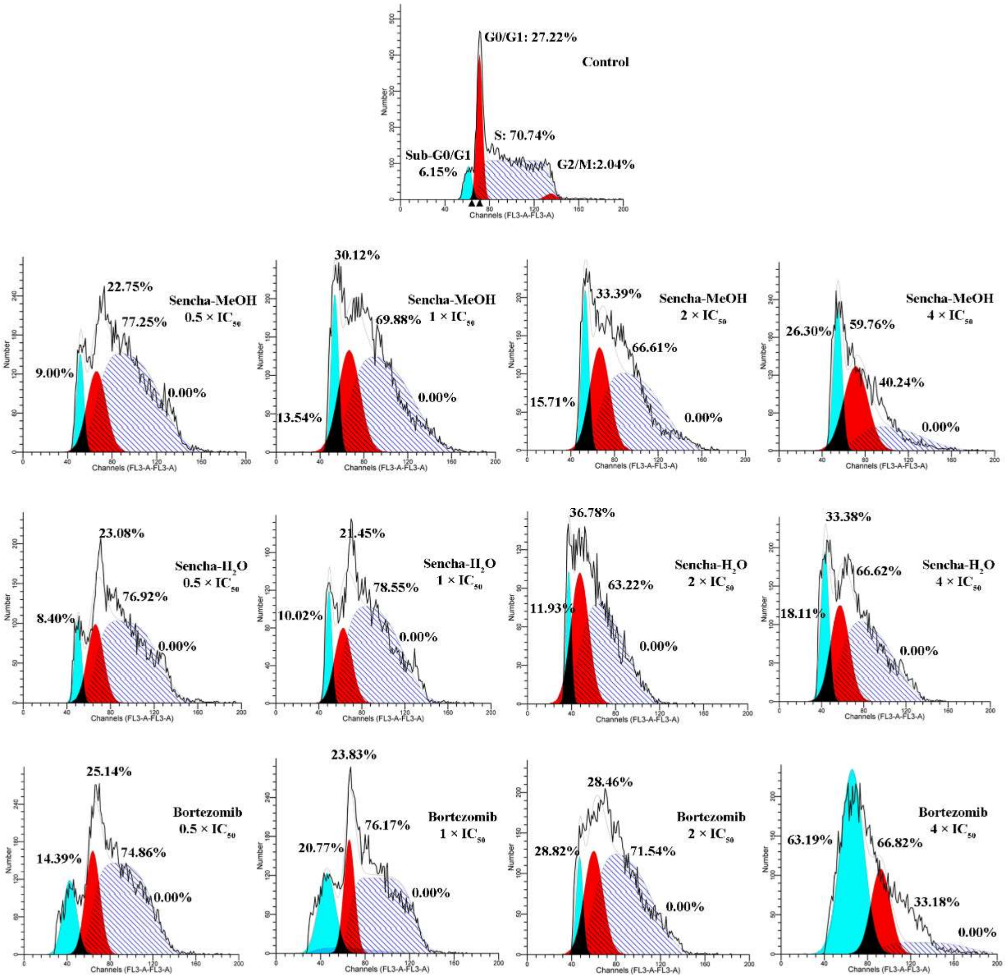

Figure S6. Distribution of cell cycle of KMS-12-BM upon 72 h treatment with sencha-MeOH extract, sencha-H_2O extracts and bortezomib at a set of concentrations. IC_{50} values were 11.37 μg/mL for sencha-MeOH extract, 14.85 μg/mL for sencha-H_2O extract, 0.0019 μM for bortezomib towards KMS-12-BM cells.
